# Arteriogenesis of the Spinal Cord—The Network Challenge

**DOI:** 10.3390/cells9020501

**Published:** 2020-02-22

**Authors:** Florian Simon, Markus Udo Wagenhäuser, Albert Busch, Hubert Schelzig, Alexander Gombert

**Affiliations:** 1 Department of Vascular and Endovascular Surgery, Heinrich-Heine-University of Düsseldorf, 40225 Düsseldorf, Germany; markus.wagenhaeuser@med.uni-duesseldorf.de (M.U.W.); hubert.schelzig@med.uni-duesseldorf.de (H.S.); 2 Department of Vascular and Endovascular Surgery, Klinikum rechts der Isar, Technical University of Munich, 81675 Munich, Germany; albert.busch@mri.tum.de; 3 Department of Vascular Surgery, University Hospital RWTH Aachen, 52074 Aachen, Germany; agombert@ukaachen.de

**Keywords:** spinal cord ischemia, arteriogenesis, paraplegia, aortic disease, TAAA, collateral network, paraspinous compartment, NO, VEGF, NOTCH

## Abstract

Spinal cord ischemia (SCI) is a clinical complication following aortic repair that significantly impairs the quality and expectancy of life. Despite some strategies, like cerebrospinal fluid drainage, the occurrence of neurological symptoms, such as paraplegia and paraparesis, remains unpredictable. Beside the major blood supply through conduit arteries, a huge collateral network protects the central nervous system from ischemia—the paraspinous and the intraspinal compartment. The intraspinal arcades maintain perfusion pressure following a sudden inflow interruption, whereas the paraspinal system first needs to undergo arteriogenesis to ensure sufficient blood supply after an acute ischemic insult. The so-called steal phenomenon can even worsen the postoperative situation by causing the hypoperfusion of the spine when, shortly after thoracoabdominal aortic aneurysm (TAAA) surgery, muscles connected with the network divert blood and cause additional stress. Vessels are a conglomeration of different cell types involved in adapting to stress, like endothelial cells, smooth muscle cells, and pericytes. This adaption to stress is subdivided in three phases—initiation, growth, and the maturation phase. In fields of endovascular aortic aneurysm repair, pre-operative selective segmental artery occlusion may enable the development of a sufficient collateral network by stimulating collateral vessel growth, which, again, may prevent spinal cord ischemia. Among others, the major signaling pathways include the phosphoinositide 3 kinase (PI3K) pathway/the antiapoptotic kinase (AKT) pathway/the endothelial nitric oxide synthase (eNOS) pathway, the Erk1, the delta-like ligand (DII), the jagged (Jag)/NOTCH pathway, and the midkine regulatory cytokine signaling pathways.

## 1. Introduction

Spinal cord ischemia (SCI) is a major clinical complication of aortic repair. A complex aortic aneurysm, such as thoracoabdominal aortic aneurysm (TAAA), is a rare and potentially lethal condition. Even in experienced centers, both open and endovascular repair of, specially, type II TAAA is associated with severe complications and in-hospital mortality, evidently provoked by the replacement of the entire descending thoracic and abdominal aorta, often associated with iliac artery repair. Improved surgical techniques and protective measures have improved outcomes preoperatively and during follow up [1,2].

Back in the 1980s, about one third of the patients that underwent thoracic or thoracoabdominal aortic surgery suffered from neurological disabilities afterwards. Even with the introduction of endovascular approaches into the clinical routine, SCI remains a devastating complication that is associated with the extent of aortic replacement and/or stent graft coverage. Thoracic endovascular aortic repair (TEVAR) has undergone a tremendous evolution in past decades. In the case of a complicated aortic type B dissection, according to the Stanford classification, which is defined as a malperfusion of sprouting aortic branches resulting in, e.g., paraplegia, endovascular aortic repair seems to be favorable today, as it seems to be related to a decreased mortality rate and a reduced complication rate when compared with open aortic repair [3,4,5,6,7]. The incidence of SCI ranges from 1.2% to 8% following TEVAR and is of utmost interest since neurological dysfunctions significantly impair the quality of life, and even reduce life expectancy in the long-term [8]. The incidence of SCI following open surgical procedures for thoracic aortic aneurysm is as high as 2%–19%, which exceeds the rates seen after TEVAR. There is a clear preference for TEVAR in all major industrial countries, because of the reduced rates of paraplegia [9,10]. However, even pararenal endovascular aortic repair holds the risk of spinal cord ischemia, especially when the hypogastric artery becomes occluded during the procedure [11,12]. More specifically, there are numerous risk factors that influence the patient outcome, such as, e.g., the extended length of covered aortic segments, the placement of stent grafts between TH9-Th12, the occlusion of the left subclavian artery, perioperative hypotension, and long total procedure time [8]. Considering these risk factors, there are strategies which have proven to be capable to reduce the incidence of SCI. These strategies include cerebrospinal fluid drainage (CSFD), local spinal cord cooling, re-implantation of segmental arteries during open surgical procedures, and prevention of hypotensive episodes during and after surgery. Although all these measures follow a comprehensive physiological theory, the current literature reveals only limited clinical success [7,13,14,15]. The beneficial application of somatosensoric (SSEP) and motoric-evoked potentials (MEP) during TAAA surgery has been described before [16]. A reduction in MEP amplitude to less than 50% of the baseline is considered an indication of ischemic spinal cord dysfunction. If the signals remain normal, intercostal arteries can be reattached if the aortic wall allowed a safe anastomosis during open TAAA repair. In case of a decrease, patent intercostal or lumbar arteries are revascularized. Even when applying somatosensoric and motoric-evoked potentials during surgery to identify relevant segmental arteries to maintain a sufficient blood supply to the spinal cord, paraplegia is not preventable for all cases and the application of these potentials is not clearly recommended according to the current guidelines of the European Society for Vascular Surgery (ESVS) [7,17].

As a perspective, the application of biomarkers, which can be measured in patients’ blood and cerebrospinal fluid (CSF), could be a further option to detect spinal cord ischemia postoperatively. These could be a possibility to monitor the spinal cord function pre-, intra-, and postoperatively. Based on the experience in the fields of traumatology, several biomarkers have been assessed which could be associated with acute spinal cord trauma [18]. Elevated levels of lactate in the CSF as well as elevated levels of neurone-specific enolase (NSE), glial fibrillary acidic protein (GFAP), and S100B in CSF and serum have been assessed as promising biomarkers to monitor acute spinal cord damage due to ischemia [19,20,21]. In fields of complex aortic surgery, only a few studies evaluated the applicability of biomarkers to detect spinal cord ischemia. Regarding S-100β in the CSF, ambiguous results could be observed in the existent studies, as levels of S-100β were not significantly higher in some studies for patients who suffered from SCI compared to the control group [22]. NSE, a dominant enolase-isoenzyme found in neuronal and neuroendocrine tissues, is a 78 kD gamma-homodimer. The biological half-life of NSE in body fluids is approximately 24 h. NSE levels in CSF were measured in the study of Lases et al. and have been compared with standard MEP monitoring. The authors found a poor correlation between CSF levels of NSE and postoperative paraplegia, although patients suffering from SCI had greater levels of NSE than the 90th percentile of patients with no adverse neurological outcomes. GFAP, an intermediate filament protein expressed by many cell types of the central nervous system, was first described in 1971 [23]. GFAp, which it was first named, isolated and characterized by Eng et al. in 1969, is estimated to maintain astrocyte mechanical strength [24]. In their study, Anderson et al. reported GFAP measurements in 11 patients that underwent complex open TAAA repair [25]. Only a rather weak correlation of biomarker levels and clinically relevant endpoints, such as SCI, could be observed; only one patient suffered from SCI. In this case, a significant elevation of biomarker levels could be assessed. This finding is typical for studies focusing on biomarkers and SCI in fields of aortic surgery. No study leading to clear results which would support a recommendation for the routine application of biomarkers has been conducted so far.

As described above, there is a wide range of established factors which may predict a patient’s risk for spinal cord ischemia. However, we are unable to predict which patient will develop postoperative problems. One possible reason for this issue is the rather unknown arteriogenesis of the spinal cord blood supply, because the loss of a single segmental artery probably causes maturation of the paraspinal collaterals, which might be a fostering condition for patients undergoing therapy of an aortic disease [26,27]. The sweeping relevance of these complications and the lack of treatment options make it worth studying every possibility to increase the positive outcome of a patient’s quality of life. Therefore, this review aims to illuminate arteriogenesis in general, with the focus on the special needs of the spinal cord blood supply.

## 2. Blood Supply of the Spinal Cord

Most of what is known today about the arterial supply to the spinal cord goes back to some studies from the last century [28,29,30,31].

When entering the medulla, various branches are sprouting from the vertebral arteries that merge to form the anterior spinal artery (ASA). The ASA courses midline on the ventral sulcus of the spinal cord and merge with approximately 10–12 segmental arteries, which arise from various branches of the aorta. These segmental arteries are known as medullary arteries. Furthermore, paired posterior spinal arteries (PSAs) arise from the vertebral arteries or the posterior inferior cerebellar artery adjacent to the medulla oblongata and course on the surface of the spinal cord medial to the posterior root entry zone. The ASA gives rise to numerous sulcal branches that supply the anterior two thirds of the spinal cord. The PSAs supply much of the dorsal horn and the dorsal columns. A tightly organized network of vessels, known as the vasocorona, connects these two sources of supply and sends branches into the white matter around the margin of the spinal cord [32].

So far, this is the doctrine of the blood supply of the spinal cord, but there is emerging evidence of a huge collateral network protecting the central nervous system from ischemia [33]. However, the structure and functionality of this network might be very different than initially thought. For instance, there is a network in close relation to the spinal cord—the paraspinous and the intraspinal compartment. The paraspinous vessels are small, nonconducting arterioles, whereas the intraspinal system consists of circle- or pentagon-shaped small conducting arteries. These arteries connect adjacent segments [34,35]. It is noteworthy that the anatomical structure of the vessel system of paraspinous and intraspinal arteries accounts for their disproportionate impact in restoring the blood flow in cases of an acute interruption of the segmental inflow. In more detail, the intraspinal arcades are essential to maintain blood pressure immediately after blood inflow interruption. Without these arcades, the blood flow would almost drop to zero and the perfusion pressure would not recover, which, in turn, does not allow reactive hyperemia, resulting in a severe ischemia of the spinal cord with following paraplegia [36]. On the other hand, the paraspinal system of immature nonconducting arterioles needs to undergo arteriogenesis to ensure ongoing blood flow after the acute ischemic insult [36]. The emergency system of epidural arcades of the intraspinal system remains functional if the anterior radiculomedullary arteries (ARMAs) are sufficiently established to ensure blood flow to the anterior spinal artery (ASA). All these vessels are closely related to each other and are connected via longitudinal anastomoses. In case of suddenly losing parts of the segmental inflow, there is a repetitive ring-shaped arterial pattern on the dorsal surface of the vertebral bodies, which, until recently, remained unnoticed. These arterial vessels might be part of a stopgap to ensure blood supply to the spinal cord [34].

The collateral network does not consist only of the blood vessels directly surrounding the spinal cord, but also includes segmental arteries, the subclavian and/or iliac arteries, the aforementioned vessels of the central nervous system, the vessels of the paraspinous muscles, and the vessels of other paravertebral tissues [33,34,37]. The entirety of this vessel system merges with the internal thoracic, epigastric, intercostal, and lumbar arteries to form a network that can be filled even from distant inputs. The reason for this is so that the network ensures a redistribution of the blood volume as long as the blood pressure as driving force is high enough [34]. The varying roles of such major vessels are of specific interest in modern (endo)vascular surgery, where the ambition is to preserve as many vessels connected to the lumbar feed as possible. However, this dogma is challenged by specific surgical approaches needing specific coverage of such vessels.

As well as blood pressure being one of the most important forces to keep up the perfusion of the spinal cord, the radius of the arteries is also of special importance. In particular, in the vessels suffering from chronic ischemia caused by increasing stenosis of the feeding arteries, the arterial radius is a powerful driver of pressure drop across the stenosis. This significance is described when considering Poiseuille’s law. Here, Poiseuille stated that the fourth power of the radius of an artery is reciprocally associated with the pressure, as shown below:

Poiseuille’s law “Pressure drop across stenosis = (blood flow × 8Lη)/πr4”, where L is the length of stenosis, η is viscosity, and r is the radius of the artery [38].

**Figure 1:**
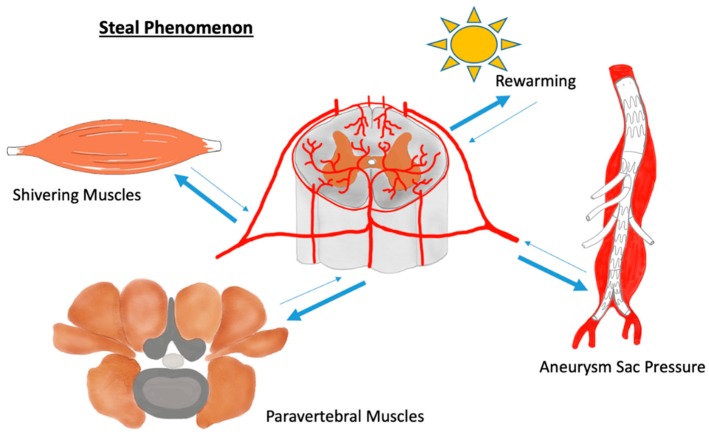
During a steal phenomenon blood becomes redistributed endangering spinal cord blood supply by hypoperfusion.

This may underline the flexibility of the blood vessel system, resulting in the ability to re-distribute significant blood volumes through longitudinal artery anastomoses. In fact, there should not be too much fear of losing one segmental artery or of the artery of Adamkiewicz, because these vessels may be appropriately compensated by the spinal cord network [13,39,40,41]. In contrast, the paravertebral muscles not only ensure blood supply to the spinal cord but can also endanger the central nerves by so-called steal phenomena. During a steal phenomenon, blood is redistributed by alternate routes or reversed flow, causing hypoperfusion in the vessel bed from which blood is withdrawn. That said, the steal phenomena can cause considerable hypoperfusion of the spine when muscles that are connected with the network bypass blood. This may happen during body movements and may be of particular importance during the first 24–72 h after TAAA surgery [17,42,43]. In this regard, the delayed rewarming and shivering of patients might cause steal phenomena. However, not only the musculature can endanger the blood supply of the spinal cord via a steal phenomenon. Additionally, or especially, the intestinal aortic passages can lead to a critical undersupply of blood to the spinal cord during endovascular TAAA surgery. It was observed that patients undergoing such an intervention showed a collapse of the motor evoked potentials (MEP) during the procedure and necessary but temporary balloon occlusion of the aorta. The reason for this is the reduced pressure in the aneurysm sac during vessel cannulation, which, due to the temporary pressure gradient, withdraws blood retrogradely from the spinal cord blood network [44] (Figure 1). Last but not least, the venous system can also contribute to reduced arterial perfusion via an elevated venous pressure and/or the expansion of the venous system [17]. For these reasons, therapists have established measures during and after surgery, such as, e.g., intraoperative hypothermia and the use of relaxants during the first postoperative hours to reduce the metabolic demands of the paraspinal muscles [45,46].

Although little is known about arteriogenesis in the vessel network of the spinal cord, it seems very likely that findings from other locations in the body are transferable, since the arteries of the spinal cord and collateral arteries of the extremities both originate from skeletal muscle arterioles. This means, in particular, that findings that mostly concerned arteries of the lower extremities should also be true for other arteries of same origin but different anatomical position [47,48]. That being said, it is the paraspinal collaterals that most likely undergo arteriogenesis during chronic thoracic or thoraco-abdominal aortic diseases. In particular, the typical corkscrew formation, which is well known from collaterals in peripheral arterial occlusive disease, can also be observed for TAAA [26,49]. Patients with extensive aortic disease often form large arteriogenic collaterals in the paraspinous region, which connect adjacent segmental arteries in the case of isolated segmental occlusion [35].

## 3. Stress-Related Changes in Blood Vessels

Due to the highly adaptive nature of the vascular system connecting all organs and systems in the body, it is a landmark for the progression and prevention of diseases. Vessels are a conglomeration of different cell types and consist of more than just endothelial cells (ECs). Each of them is involved in adapting to stress, ultimately resulting in vessel formation during arteriogenesis, like smooth muscle cells and pericytes [50,51]. Pericytes, for example, encompass endothelial cells and contribute to vessel integrity [52]. To this end, arteriogenesis is defined as dilation and remodeling of pre-existing small arteries or capillaries into vessels that can foster more blood volume [53,54]. This kind of vessel adaptation follows flow volume alterations and can also be observed in the paraspinal network. Here, the immature collaterals dilatate and increase in length to meet the elevated demand of blood volume of the spinal cord. Moreover, the pre-existing unstructured arterioles react with parallel realignment [55]. Again, these arteriogenic adaptions are somewhat comparable to observations in other tissues [35].

The phenotype of the vasculature of each organ is not a given, inflexible, or even unadaptable situation after embryogenic maturation. It is more a vivid system that might change depending on, e.g., metabolic needs, oxygen availability, oxygen radicals, and shear stress [56,57,58,59,60]. Under pathological conditions, like chronic ischemia, the identifying markers of the vessel walls change, indicating the convertibility of this biological system [61]. More specifically, enhanced shear stress might result in arteriogenesis, which establishes a biological bypass to circumvent the slowly growing stenosis of a vessel [62,63,64]. During this adaption, the collateral vessels dilatate to compensate for the reduced blood flow through a stenosis in a vessel [65]. As far as we know, a vessel’s adaption can be described as follows:

During initiation, the local endothelium gets activated by the enhanced shear stress, resulting in the recruitment of local and bone marrow inflammatory cells [66,67]. These cells release several chemokines, e.g., tumor necrosis factor (TNF) and vascular endothelial growth factor (VEGF). VEGF is an important factor for vessel formation during angiogenesis. However, the expression level decreases during the late embryonic phases. This observation is given particular interest, since the mature central nervous system only expresses very low levels of VEGF to prevent blood–brain barrier leakage. In contrast, fenestrated capillaries release high VEGF levels. Such vessels may be found in the kidneys. Inflammatory cells are recruited and bind to the surface of the endothelial cells through various adhesion molecules, such as selectins, intercellular adhesion molecule 1 (iCAM1), and vascular adhesion molecule 1 (vCAM1). Following transmigration, it is the neutrophil cells that degrade the extracellular matrix to create space for expanding vessels. The recruitment of circulating monocytes paves the way for the next step of arteriogenesis [63,68,69,70,71,72,73,74].

During the growth phase, macrophages recruit other bone marrow-derived cells, vascular smooth muscle cells, and endothelial cells. Several chemokines, like TNF, VEGF, fibroblast growth factor (FGF), platelet derived growth factor (PDGF), granulocyte macrophage-colony stimulating factor (GM-CSF), monocyte chemoattractant protein-1 (MCP-1), and transforming growth factor are released, amongst others, by macrophages and the smooth muscle cells coordinate these actions [75]. matrix metallopeptidases 2 (MMP-2) and 9 (MMP-9) contribute to the remodeling of the basement membrane. Following a reversible shift along a continuum from a quiescent, contractile phenotype to a synthetic phenotype, vascular smooth muscle cells (VSMC) start to migrate and proliferate. The sequence of extracellular matrix (ECM) degradation and altered differentiation towards the synthesis and proliferation in the cells of the vessel wall contribute to the “new” vessels with their typical tortuous elongation and increased overall cross-section. Comprehensively, all these adaptive alterations ultimately decrease the local resistance of the vasculature, aiming to restore blood flow, as described by Poiseuille’s law [38,63,66,74,76,77,78,79,80].

During the last phase, the so-called maturation, all processes characterizing arteriogenesis return to normal levels. That said, the shear stress decreases because the blood flow is distributed through the collateral network, causing pressure, flow, and stress reduction to the vessels. Likewise, the endothelium function normalizes, and the inflammatory activity gets downregulated. Furthermore, cell proliferation declines and the phenotypic shift of the vascular smooth muscle cells revokes. During maturation, the fate of the newly formed collaterals is different. While the collaterals at high flow rates stabilize, smaller collaterals at low flow rates regress [63,74,81,82]. To get an idea of the dimensions in which arteriogenesis may alter collateralization, experimental data in pigs were applied. Five days after the occlusion of all segmental arteries, vessels from different anatomical structures grew significantly. In more detail, the epidural arcades expanded from 150 to 249 μm. Likewise, an increase in diameter was observed for the ASA, ranging from 90 to 137 μm [55].

After considering all the aforementioned aspects, one should not miss one of the most significant determinates of tissue survival and cell death. This factor is the timespan. Due to its significance, there might be a huge difference in outcome depending on how the timespan within the alterations is established and how many pre-existing collaterals already exist. In fact, different tissues take different times to adapt. In animals, there is evidence that arteriogenesis in ischemic tissue is fast in the nervous system, where it takes only three days to reach the maximum of collateral remodeling, while it takes up to seven days in hearts, or even up to four weeks in skeletal muscles. This observation can be translated to what is seen in patients and correlated to their outcome. Here, the more pre-existing collaterals with the appropriate capability of remodeling, the better the clinical outcome [62,67,74,83,84,85,86]. Experimental findings also support this paradigm. In these settings, recent experimental data suggest that the blood supply to the spinal cord is mainly dependent on a well-established feeding vessel network and on its subsequent improvements via arteriogenesis [87,88]. Of note, there is data suggesting that these beneficial adaptions could be mimicked prior to surgery in the form of pre-conditioning the blood supply to the spinal cord. Preconditioning, in this context, means that segmental arteries become occluded by, e.g., coil embolization, to stimulate collateral network growth. Anatomical and physiological knowledge of the spinal cord circulation could improve open and endovascular TAAA repair by enabling or promoting a staged procedure that could improve spinal cord protection. Furthermore, preconditioning of the spinal cord before open or endovascular TAAA repair could result in the reduced vulnerability of the spinal cord during the first postoperative days. Animal studies, conducted in 2015 by the same research group, underline the beneficial application of the pre-operative embolization of intercostal and lumbar arteries before TEVAR; pigs with pre-interventional coil-embolization before TEVAR suffered significantly less frequently from SCI than the control group [89,90]. Moreover, there is clinical evidence that pre-conditioning enhances arteriogenesis, improving the blood supply to the central nervous system. That being said, such pre-conditioning has the potential to reduce the risk of paraplegia or paraparesis after TEVAR and might, therefore, be integrated into the clinical routine [91,92].

In the central nervous system (CNS), the barrier between the bloodstream and the underlying tissue is built up of special ECs that are less thick compared to the ECs of skeletal muscle. In addition, the blood–brain barrier (BBB) controls the passage of selected substances, such as ions, etc., into the brain. Hence, the ECs of the central nervous system (CNS) are continuous and non-fenestrated, with only a few exceptions [93]. For these reasons, the adaptions in this vascular bed are of particular interest, since they differ from other locations. In the case of an ischemic insult or chronic worsening ischemia caused by a severe narrowing of the feeding arteries of the spinal cord, the disruption of the BBB is unfavorable since its consequences, such as spinal edema or superinfections, are severe. Arteriogenesis serves as biological bypass for such stenoses and effectively maintains the blood–brain barrier since it is “only” an expansion of pre-existing collaterals.

## 4. Signaling Pathways

There are some important signaling pathways that contribute to arteriogenesis. For instance, the activation of the phosphoinositide 3 kinase (PI3K)/antiapoptotic kinase (AKT)/endothelial nitric oxide synthase (eNOS) pathway plays a major role in the remodeling of the collaterals. The PI3K/AKT pathway has an impact on many processes, like metabolism, apoptosis, cell survival in general, and cell proliferation. The most important effects during arteriogenesis are cell proliferation, cell growth, and reduction in apoptosis. PI3K is an intracellular signal transducer that activates the downstream target AKT that is able to phosphorylate eNOS. The activated eNOS is responsible for an increase of NO expression, which ameliorates cell survival, among other things. NO is a landmark effector and its production causes vasodilatation that enhances the blood flow, which, in turn, stabilizes vessel remodeling effects. The pathway also maintains interactions between ECs and other cell types of the surrounding tissue, such as VSMC and pericytes. Both cell types are significantly involved in the promotion and maintenance of ateriogenesis and its stabilization. In this regard, there are different sources of NO. The endothelial nitric oxide synthase (eNOS), neuronal nitric oxide synthase (nNOS), and the inducible nitric oxide synthase (iNOS) contribute to arteriogenesis and can replace each other’s production to meet the demand of the required NO levels [94,95,96,97,98,99,100,101].

The extent of arteriogenesis is directly linked to the activation of the extracellular signal-regulated kinase 1 and 2 (ERK1/2) pathway. The two isoforms have different roles, but work hand-in-hand in arteriogenesis. VEGF is an important signal molecule for the activation of the endothelial ERK1/2, pathway resulting in increased arteriogenesis or, rather, the absence of VEGF causes a reduction in arteriogenesis. As mentioned above, inflammatory cells are necessary for arteriogenesis, especially as these cells are a major source of VEGF in absence of tissue ischemia. ERK1 is a key holder of macrophage infiltration. When ERK1 is absent, it creates a massive macrophage infiltration with excessive VEGF expression. This oversupply does not result in improved functional vessels—quite the opposite. The seemingly improved arteriogenesis is built up of only poorly functional vessels that do not increase blood flow sufficiently. In contrast to the effects of ERK1, the loss of the endothelial ERK2 pathway does not affect arteriogensis at first sight, but does end in reduced blood flow recovery. The reason for the diminished function of arteriogenesis is the positive stimulation of endothelial cell proliferation and eNOS expression that fails in the absence of ERK2. In particular, the missing NO production results in vasoconstriction with the aforementioned reduced blood flow [102,103,104,105,106].

Another important pathway is the delta-like ligand (DII) and jagged (Jag)/NOTCH pathway that is responsible for perivascular macrophage maturation and the inflammatory response, resulting in the remodeling of the newly formed collaterals. NOTCH is a family of transmembrane proteins that get cleaved following ligand binding. After such cleavage, the intracellular domain is translocated into the nucleus. NOTCH signaling is also of particular significance in the close interaction between ECs and VSMCs. Here, cytokines, such as VEGF and FGF, enhance the expression of DII, which, in turn, elevates EPHB-2/4 plasma levels by NOTCH signaling. The aforementioned process is essential for the precise coordination of vessel remodeling during arteriogenesis [54,74,107,108,109,110,111,112,113,114]. VEGF signaling improves EC survival, because the above-mentioned phosphoinositide 3 kinases (PI3Ks) and antiapoptotic kinases (AKTs) are increased by VEGF. Additionally, the elevation of NO levels, caused by vascular endothelial growth factor receptor 2/neuropilin 1 (VEGFR-2/NRP-1), influence lumen expansion, vascular remodeling, and maturation/stabilization [115]. Moreover, VEGF also stimulates other receptors, such as the erythropoietin-producing hepatocellular (Eph) receptor. This tyrosin kinase is categorized into two subtypes—Eph-A and Eph-B. Those two receptors are bound to ephrin-A and ephrin-B. Ephrin-B, for example, internalizes via endocytosis VEGFR-2 and prevents PDGF endocytosis. Thus, it contributes to VSMC maturation, which plays a key role in stabilizing the arterial wall. These ligands and receptors are not only found on vessels where they serve as identification marker, but are also found in the nervous system [116,117,118,119,120,121]. Tumor growth factor ß (TGF-ß) cytokine is also involved in arteriogenesis. Interestingly, the expression of this cytokine is stimulated in hypoxic conditions through HIF-1α and oxidative stress. TGF-ß up-regulates collagen and produces and promotes vascular remodeling, although it is not the primary effector in arteriogenesis [122]. Macrophages are inflammatory cells and also contribute to the remodeling of the vessel wall. During arteriogenesis, macrophages increase the number of VSMCs within the arterial wall, which stabilizes the newly formed vessel [74,123,124].

A midkine (MK) is a regulatory cytokine during embryonic vessel angiogenesis, and its dysfunctional signaling causes malignant diseases. Meanwhile, rising evidence has linked this cytokine to arteriogenesis via, e.g., elevated VEGF-A levels [94,125,126,127]. The positively charged molecule binds to the endothelium, where several receptors/receptor complexes interact with MK and mediate its downstream signaling. Due to its high significance for key processes, it is worth mentioning that the endothelium itself might be a relevant source of MK [128,129]. Interestingly, MK interferes with the above-mentioned NOTCH receptor, suggesting significant potential for altering the inflammatory response via iCAM1. This is further supported by animal experiments, since MK-deficient mice revealed reduced leucocyte infiltration, which has a severe impact on arteriogenesis [130,131,132,133].

## 5. Conclusions

The feeding vessel network to the spinal cord is well known. Since SCI is a persisting clinical problem after open and endovascular aortic procedures, the scientific community has focused, once again, on the paraspinous and the intraspinal compartment. The aortic intervention-related steal phenomena can cause the hypoperfusion of the spine since large-volume muscles connected with the network and body movements can shift significant blood volumes shortly after TAAA surgery, which may be of importance during the first 24–72 h postoperatively. Even delayed rewarming and shivering might enhance the steal phenomena which contribute to SCI. Pre-conditioning with selective segmental artery occlusion reduces the blood supply to the CNS artificially, enhancing arteriogenesis. Both, arteries of the spinal cord as well as of the extremities are of skeletal muscle arterioles origin with transferable mechanisms according to arteriogenesis, involving signaling pathways like PI3K/AKT/eNOS, Erk1, the delta-like ligand, jagged (Jag)/NOTCH pathway, and the Midkine regulatory cytokine signaling. In the future, both arteriogenesis enhanced by the preconditioning of the blood supply of the central nervous system via selective segmental artery occlusion and the validation of biomarkers, e.g., NSE, GFAP, and S100B, might become additional cornerstones in the treatment of elective thoracic aortic repair, leading to a reduced risk of paraplegia or paraparesis.
